# A ‘Disease Severity Index’ to identify individuals with Subjective Memory Decline who will progress to mild cognitive impairment or dementia

**DOI:** 10.1038/srep44368

**Published:** 2017-03-13

**Authors:** Daniel Ferreira, Farshad Falahati, Cecilia Linden, Rachel F. Buckley, Kathryn A. Ellis, Greg Savage, Victor L. Villemagne, Christopher C. Rowe, David Ames, Andrew Simmons, Eric Westman

**Affiliations:** 1 Division of Clinical Geriatrics, Centre for Alzheimer Research, Department of Neurobiology, Care Sciences and Society, Karolinska Institutet, 141 86 Stockholm, Sweden; 2 Melbourne School of Psychological Sciences, University of Melbourne, Melbourne 3010, Australia; 3 The Florey Institute of Neuroscience and Mental Health, University of Melbourne, Melbourne 3084, Australia; 4 Department of Neurology, Massachusetts General Hospital/Harvard Medical School, 02129 Boston, MA, USA; 5 The Academic Unit for Psychiatry of Old Age, University of Melbourne, Melbourne 3052, Australia; 6 ARC Centre of Excellence in Cognition and its Disorders, Department of Psychology, Macquarie University, Sydney 2109, Australia; 7 Department of Nuclear Medicine and Centre for PET, Austin Health, Heidelberg 3084, Australia; 8 Department of Medicine, Austin Health, University of Melbourne, Melbourne 3084, Australia; 9 NIHR Biomedical Research Centre for Mental Health, London SE5 8AF, UK; 10 NIHR Biomedical Research Unit for Dementia, London SE5 8AF, UK; 11 Department of Neuroimaging, Centre for Neuroimaging Sciences, Institute of Psychiatry, Psychology and Neuroscience, King’s College London, London SE5 8AF, UK

## Abstract

Subjective memory decline (SMD) is a heterogeneous condition. While SMD might be the earliest sign of Alzheimer’s disease (AD), it also occurs in aging and various neurological, medical, and psychiatric conditions. Identifying those with higher risk to develop dementia is thus a major challenge. We tested a novel disease severity index generated by multivariate data analysis with numerous structural MRI measures as input. The index was used to identify SMD individuals with high risk of progression to mild cognitive impairment (MCI) or AD. A total of 69 healthy controls, 86 SMD, 45 MCI, and 38 AD patients were included. Subjects were followed up for 7.5 years. Clinical, cognitive, PET amyloid imaging and APOE ε4 data were used as outcome variables. The results showed that SMD evidenced cognitive performance intermediate between healthy controls and MCI. The disease severity index identified eleven (13%) SMD individuals with an AD-like pattern of brain atrophy. These individuals showed lower cognitive performance, increased CDR-SOB, higher amyloid burden and worse clinical progression (6.2 times higher likelihood to develop MCI, dementia or die than healthy controls). The current disease severity index may have relevance for clinical practice, as well as for selecting appropriate individuals for clinical trials.

The pathophysiological process of Alzheimer’s disease (AD) is believed to begin years before clinical symptoms become apparent^1^. This makes the preclinical phase a conceivable opportunity for early detection and intervention^2^. Cumulative evidence indicates that complaints of subjective memory decline (SMD) in cognitively normal older adults are a risk factor for future progression to mild cognitive impairment (MCI) or the dementia stage of AD (see ref. 3 for a review and meta-analysis). However, SMD is not specific to AD but also occurs in aging and various neurological, medical, and psychiatric conditions. Substance use and personality traits are also associated with SMD^4^. Therefore, SMD is a heterogeneous condition. In addition, the association between SMD and objective cognitive performance has yielded varying results^5^. Recent reports show a nuanced relationship between these that is difficult to capture in cross-sectional studies and heterogeneous samples^6^,^7^. Hence, a more consistent association between SMD and objective cognitive tests is found in longitudinal studies or more homogeneous samples, for example in those with high β-amyloid burden or APOE ε4 carriers^6^,^8^,^9^,^10^,^11^,^12^,^13^,^14^. Importantly, if a combination of subjective complaints and biomarkers is desired to be included in routine screening for dementia, the reality is that information about β-amyloid and APOE status is not always available in clinical settings. Structural magnetic resonance imaging (MRI) is widely available and allows measuring downstream neurodegeneration, another key hallmark of AD. Moreover, neurodegeneration correlates with cognitive decline and disease progression more strongly than β-amyloid and APOE status do^15^.

AD is typically characterized by atrophy primarily involving the medial temporal lobes^16^, a pattern that has also been described in healthy adults with subjective complaints^17^,^18^,^19^,^20^,^21^. Studying patterns of atrophy instead of single regions of interest has yielded higher diagnostic performance^16^. These patterns of atrophy can be captured with multivariate data analysis methods such as *Orthogonal Projection to Latent Structures* (OPLS)^22^. By building models on multiple structural MRI measures from AD patients and healthy controls, AD patients can be discriminated from the healthy controls with sensitivity and specificity values around 90%^23^. New and unseen data can also be projected onto this model and a ‘disease severity index’ can be created for each individual subject. For example, we have used this index to predict progression from MCI to AD in two previous studies^24^,^25^. The disease severity index describes if an individual has an AD-like pattern of brain atrophy or a healthy control-like (HC-like) pattern of brain atrophy. To our knowledge, this method has not been applied to SMD individuals yet. However, it has previously been proposed that cognitively normal individuals with an AD-like pattern of brain atrophy could be considered to have preclinical AD^19^,^25^. Since only mild neuronal damage is expected in SMD^17^,^18^, identifying those individuals who will possibly progress to MCI and AD is of utmost importance for early and effective intervention.

The first aim of the current study was to characterise a group of SMD individuals and test whether those with SMD exhibited cognitive performance intermediate with healthy controls and MCI/AD patients. The second aim was to investigate whether SMD individuals manifesting signs of AD could be discriminated from SMD individuals with other possible aetiologies by applying the above-mentioned disease severity index. We thus aimed to extend this method for the first time to the earlier stage of SMD. The third aim was to investigate whether the two SMD subtypes defined (AD-like SMD and HC-like SMD) differed according to relevant clinical measures at baseline as well as disease progression over 7.5 years. The second and third aims are thus addressed to validate the disease severity index in SMD. We hypothesized that SMD with an AD-like pattern of brain atrophy would have worse cognitive performance at baseline, increased neocortical amyloid burden, higher frequency of the APOE ε4 allele, and higher rate of progression to MCI or dementia.

## Results

Table 1 shows the characteristics of the study groups. SMD individuals were comparable to the healthy controls with respect to all the demographic and clinical variables. Table 2 shows cognitive performance across study groups. MANOVA clearly showed a gradual increase in cognitive impairment according to more advanced disease stages (F_(42, 540)_ = 6.149; p < 0.001; AD > MCI > SMD > healthy controls). Follow-up analyses showed that SMD performed significantly worse than healthy controls in Stroop-colours (U = −25.267; p = 0.036) and category fluency (t_(152)_ = 4.030; p < 0.001). All these results were largely consistent after accounting for the effects of age, gender, education level and the Hospital Anxiety and Depression Scale (HADS-D).

### Multivariate classification of SMD individuals based on patterns of brain atrophy

**Figure 1:**
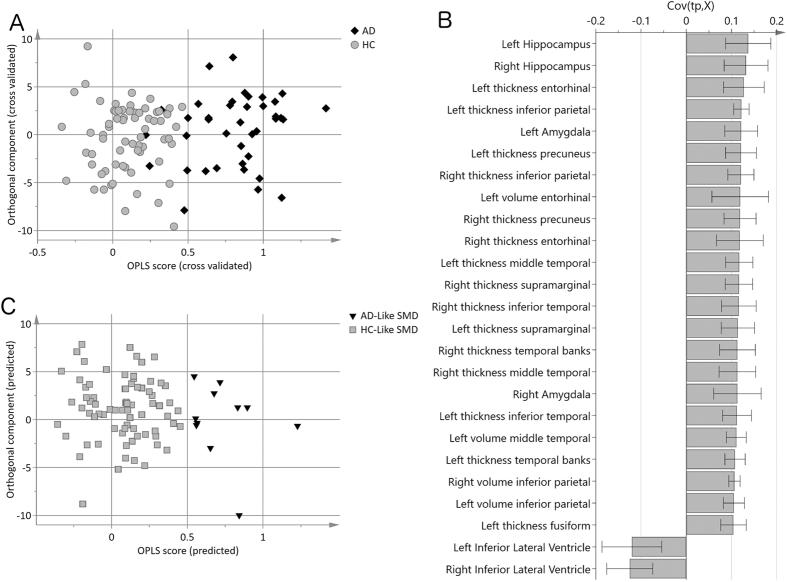
Multivariate classification of SMD individuals based on patterns of brain atrophy. (**A**) Cross-validated scores of Alzheimer’s disease (AD) patients versus healthy controls (HC). Cross-validated predictability Q2(Y) was 0.72 and sensitivity and specificity values were 84% and 100%, respectively. (**B**) Loading plot of the twenty-five most important variables for AD versus HC classification. A measure with a high covariance (y-axis) is more likely to have an impact on group separation than a measure with a low covariance. Measures above zero have a larger value in controls, including hippocampus, entorhinal cortex, inferior parietal cortex, amygdala, precuneus, etc. (i.e. reduced volume or thickness in the AD group), and measures below zero have a lower value in the controls including the lateral ventricles (i.e. larger volume in the AD group). (**C**) Prediction of SMD individuals. HC = healthy controls; SMD = subjective memory decline; AD = Alzheimer’s disease; HC-like SMD = SMD individuals evidencing a healthy-like pattern of brain atrophy; AD-like SMD = SMD individuals evidencing an AD-like pattern of brain atrophy.

An OPLS model was created to separate AD patients from the healthy controls. The model achieved a cross-validated predictability with a Q^2^(Y) value of 0.72. This model is thus regarded as significant and showed high sensitivity (84%) and specificity (100%) (Fig. 1A). The most important variables for classification were hippocampus, entorhinal cortex, inferior parietal cortex, amygdala, and precuneus, displaying reduced thickness/volume in the AD group, as well as the inferior part of the lateral ventricles, displaying larger volume in the AD group (Fig. 1B).

SMD individuals were then projected onto this AD *vs.* healthy controls model and a discriminant index was generated for each individual with SMD (i.e. ‘disease severity index’). Eleven (13%) SMD individuals were classified as having an AD-like pattern of brain atrophy (i.e. AD-like SMD subtype), and seventy-five SMD individuals were classified as having a HC-like pattern (i.e. HC-like SMD subtype) (Fig. 1C).

### Clinical characterization of the SMD subtypes

The AD-like and HC-like SMD subtypes did not differ in any of the demographic and clinical variables apart from the Clinical Dementia Rating–Sum of Boxes (CDR-SOB), where AD-like SMD scored significantly higher (χ^2^_(1)_ = 10.948; p = 0.008) (Table 3). Although MANOVA showed no global differences in cognition (F_(14,59) = _1.500; p = 0.140), significant differences were found in a number of specific cognitive tests (Table 4). AD-like SMD evidenced worse cognitive performance on the Rey Complex Figure Test (RCFT) delayed recall, Digit Symbol, and Stroop-words and -interference. Only the RCFT delayed recall effect remained significant when controlling for multiple comparisons and the effects of age, gender, education level and HADS-D (U = 228.500; p = 0.040).

**Figure 2:**
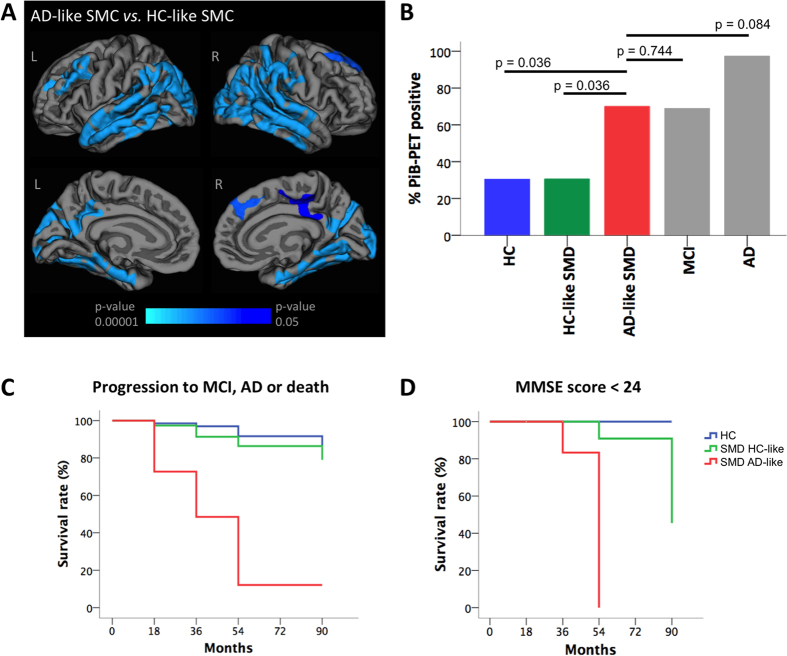
Clinical characterization of the SMD subtypes. (**A**) The cortical maps from the vertex analysis shows that AD-like SMD had reduced thickness in the medial and lateral temporal, frontal and parietal cortices as compared with HC-like SMD. Lateral surfaces are displayed in the upper row and medial surfaces are displayed in the bottom row. L = left, R = right. (**B**) ANCOVA was performed in order to compare the PiB-PET retention in AD-like SMD versus the other study groups. Benjamini-Hochberg’s corrected p-values are displayed in the figure. Age, gender, education level and HADS-D where included as covariates. (**C**,**D**) Longitudinal progression of SMD subtypes. In C, outcomes were categorized as stable if individuals remained in the same diagnostic group during the follow-up, or progressive if they developed MCI or dementia or died at the follow-up. In D, performance in MMSE was categorized as normal (≥24) or abnormal (<24). Survival was considered when SMD individuals remained stable (**C**) or showed a MMSE score ≥24 (**D**). HC = healthy controls; SMD = subjective memory decline; MCI = Mild Cognitive Impairment; AD = Alzheimer’s disease; HC-like SMD = SMD individuals evidencing a healthy-like pattern of brain atrophy; AD-like SMD = SMD individuals evidencing an AD-like pattern of brain atrophy; PiB-PET = Pittsburgh compound B–positron emission tomography.

Cortical maps of reduced thickness in AD-like SMD compared to HC-like SMD are shown in Fig. 2A to illustrate the pattern of atrophy depicted by the disease severity index. Figure 2B shows that PiB-PET retention in AD-like SMD was significantly higher than that found in healthy controls (F_(1, 76)_ = 5.453; p = 0.036) and HC-like SMD (F_(1, 79)_ = 5.276; p = 0.036), and comparable to that found in MCI (F_(1, 50)_ = 0.085; p = 0.744) and AD (F_(1, 44)_ = 8.855; p = 0.084). PiB-PET retention in HC-like SMD was comparable to that in healthy controls (F_(1, 138)_ = 0.004; p = 0.957). The PiB-PET results were obtained after controlling for age, gender, education level and HADS-D.

### Clinical progression of the SMD subtypes

72.7% (n = 8) of the AD-like SMD individuals progressed to MCI, dementia or died, while only 17.6% (n = 13) of the HC-like SMD individuals and 11.8% (n = 8) of the healthy controls progressed to MCI, dementia or died (Table 5). ANCOVA showed that the rate of progression to AD was comparable between AD-like SMD and MCI (p = 0.210), and was significantly higher in both than in HC (p < 0.001) and HC-like SMD (p < 0.001). The rate of death at follow-up was significantly higher in AD than in HC and HC-like SMD (p < 0.001). Interestingly, the rate of death was statistically comparable among AD-like SMD, MCI and AD (AD-like SMD *vs.* AD p = 0.390; AD-like SMD *vs.* MCI p = 0.820; MCI *vs.* AD p = 0.180) (Table 5).

Outcomes were categorized as stable if individuals remained in the same diagnostic group during follow-up and progressive if they progressed to MCI, dementia or died. All dementia cases were probable AD. Survival analysis showed that the rate of clinical progression to MCI, AD or death was significantly higher in AD-like SMD than in healthy controls (6.2 times higher, χ^2^_(1)_ = 40.697; p < 0.001) and HC-like SMD individuals (4.3 times higher, χ^2^_(1)_ = 29.053; p < 0.001). Clinical progression in HC-like SMD was comparable to that in healthy controls (χ^2^_(1)_ = 1.158; p < 0.282) (Fig. 2C). These results were further supported by greater attainment of abnormal mini-mental state examination (MMSE) scores in the AD-like SMD subtype (χ^2^_(2)_ = 68.125; p < 0.001) (Fig. 2D).

**Figure 3:**
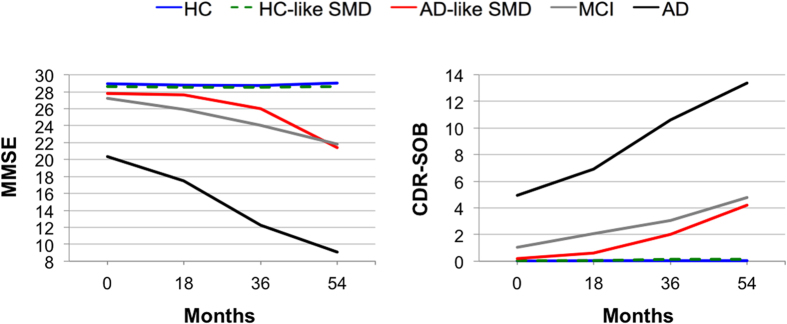
Longitudinal changes in MMSE and CDR-SOB. HC = healthy controls; SMD = subjective memory decline; HC-like SMD = SMD individuals evidencing a healthy-like pattern of brain atrophy; AD-like SMD = SMD individuals evidencing an AD-like pattern of brain atrophy; MCI = Mild Cognitive Impairment; AD = Alzheimer’s disease; MMSE = Mini-Mental State Examination; CDR-SOB = Clinical Dementia Rating–Sum of Boxes.

Table 5 and Figure 3 shows longitudinal changes in MMSE and CDR-SOB by study group. A mixed effects model was performed to investigate longitudinal changes in MMSE by study group. This model showed a significant group-by-time interaction (F_(12, 418)_ = 30.494; p < 0.001). The rate of change in AD was faster than in healthy controls in all the follow-up time points (p < 0.001). The rate of change in MCI was faster than in healthy controls at 36 and 54 months follow-ups (p < 0.001). The rate of change in AD-like SMD was faster than in healthy controls at the 54 months follow-up (p < 0.001). All the other possible pair comparisons were tested. Of interest, the rate of change in AD-like SMD was faster than in HC-like SMD at the 54 months follow-up (p < 0.001); and was statistically comparable to the one in MCI in all follow-up time points. A mixed effects model was also performed to investigate longitudinal changes in CDR-SOB by study group. This model showed almost the same results as those described above for MMSE. The only difference is that the rate of change in AD-like SMD was faster than in HC-like SMD at the 36 months follow-up in addition to the 54 months follow-up (p < 0.001). Age, gender, education level and HADS-D were included as covariates in the ANCOVA and mixed effects models described in this section.

## Discussion

The aims of the current study were to (1) characterise a group of SMD individuals and test whether they exhibited cognitive performance intermediate with healthy controls and MCI/AD patients; (2) apply a disease severity index to discriminate SMD individuals with an AD-like pattern of brain atrophy from SMD individuals with a HC-like pattern of brain atrophy; (3) investigate whether these two SMD subtypes differ according to relevant clinical measures at baseline as well as over 7.5 years. The disease severity index based on multivariate data analysis (i.e. OPLS) condenses a large amount of disease-related information in a single score and has potential diagnostic applicability^24^,^25^. This index identified a subtype of eleven (13%) SMD individuals with dramatically worse outcome (6.2 times higher likelihood to develop MCI, dementia or to die), more amyloid burden and lower cognitive performance.

As a heterogeneous group, SMD cases were clinically comparable to the healthy controls but displayed cognitive performance intermediate with the healthy controls and the MCI patients. The profile of lower cognitive performance included semantic memory (category fluency) and processing speed (Stroop-colours). The same cognitive tests correlated with subjective complaints and predicted cognitive decline or progression to dementia in several recent studies^6^,^26^,^27^. Further, this profile corresponds partially to the decline (not yet in severity) most frequently reported in mild to moderate AD^28^,^29^. These results add to the inconsistent literature on the relationship between subjective complaints and objective cognitive performance^5^. Striepens *et al*.^14^ reported worse memory performance in individuals with subjective memory impairment than in healthy controls, but they found no differences in speed/executive functions. Saykin *et al*.^18^ showed that individuals with cognitive complaints had memory performance intermediate between healthy controls and MCI when using a composite score, but not when using individual memory tests (CVLT and Logical Memory from the Wechsler Memory Scale). Amariglio *et al*.^26^ showed that some specific memory complaints but not all may indicate poor cognitive function. Other authors have found no statistical differences between individuals with subjective memory impairment and healthy controls in varied cognitive tests including CERAD, CAMCOG, trail making test, verbal fluency as well as specific episodic memory tests^17^,^19^,^20^. The relationship between subjective cognitive complaints and objective performance is not clear and the inconsistency described in previous studies may be related to sample characteristics, but also the instruments used to measure both complaints and objective performance. Our results are consistent with other studies investigating homogeneous samples with high β-amyloid burden or APOE ε4 carriers^6^,^8^,^9^,^10^,^11^,^12^,^13^,^14^, where a positive association has been described. As recently claimed, the association between subjective and objective memory do exists but is nuanced^6^. This is supported by a recent meta-analysis of 53 studies that indicates a small but reliably greater than zero association between complaints and objective memory performance^5^.

This disease profile was considerably amplified in the AD-like SMD subtype, including not only lower cognitive performance but also higher amyloid burden and worse clinical progression over 7.5 years. Previous studies have found an association between subjective complaints and amyloid burden in healthy adults^9^,^30^,^31^, including earlier reports from AIBL^11^. Chetelat *et al*.^32^ also showed convergent findings in a previous AIBL study where higher amyloid burden was related to a very similar pattern of brain atrophy to that captured by our disease severity index. Increased progression to MCI and dementia in individuals with subjective complaints is a well-established finding^3^, more prominent in those evidencing positivity for AD biomarkers^2^,^9^,^21^. A novel finding from the current study is faster cognitive decline in MMSE and clinical progression as measured by the CDR-SOB in SMD with positivity for AD biomarkers (i.e. the AD-like SMD). The fact that this result was found only at the 54 months follow-up for MMSE, but at the 36 and 54 months of follow-up for CDR-SOB suggests that CDR-SOB might be more sensitive to longitudinal changes in SMD. Another contribution of the current study is the validation of a method that allows identifying individuals under risk of clinical progression and importantly, discriminating these from a SMD subtype in which a high percentage of individuals showed stability over time. The clinical potential of this disease severity index is thus promising.

The MANOVA for cognitive variables did not show significant differences between the two SMD subtypes, indicating similar overall cognitive performance in the two groups. Nonetheless, follow-up exploratory analyses indicated lower performance in RCFT delayed recall in the AD-like SMD subtype. RCFT is a test of visual memory. Impaired recall using the RCFT has been reported in MCI^33^. In particular, RCFT delayed recall is a measure of free retrieval of visual information, a capacity that is mediated by a neural system including inferior temporal, medial temporal and frontal areas^34^. This network is consistent with the pattern of atrophy captured by our disease severity index (Fig. 2A), which highly corresponds to the one described in typical Alzheimer’s disease^35^. Atrophy in the temporal lobes has been found in heterogeneous groups of healthy adults with subjective complaints^17^,^18^,^19^,^20^,^21^ and Toledo *et al*.^36^ reported prominent frontal atrophy in SMD. In addition to the memory component, the RCFT, and specially RCFT-delayed recall, also has an important executive component^37^. Therefore, lower performance in RCFT-delayed recall in AD-like SMD may be related to difficulties in visual memory (medial temporal cortex) and perhaps difficulties in executive functions (dorsolateral cortex). These results may add to the recent literature indicating that SMD involves other cognitive functions other than memory^6^,^26^,^27^,^31^ and this is in line with early symptoms of AD not being restricted to memory alone^4^,^38^.

All these findings together with the fact that AD-like SMD evidenced increased clinical severity (i.e. higher CDR-SOB score), support the notion of AD-like SMD as truly preclinical AD. Nonetheless, the correspondence between SMD (or the broader concept of subjective cognitive decline - SCD^4^) and preclinical AD is currently under debate and still needs to be better documented. Likewise, SCD is currently postulated as the pre-MCI stage^4^, which would be supported by the results from the AD-like SMD subtype in the current study. Our findings need to be replicated in independent cohorts as well as in a larger group of SMD individuals developing AD. Nonetheless, the current results may serve as a preliminary validation of the disease severity index to discriminate between SMD with neurodegenerative aetiology versus those with possibly another cause. This is clinically relevant and to our knowledge had not been achieved in previous research. Findings in the current study still need to be carefully considered and further research is very much warranted especially regarding the HC-like SMD subtype. HC-like SMD is possibly a collection of subtypes with different aetiologies, some of them perhaps treatable (e.g. subclinical depression).

One of the main strengths of this study is tracking of the whole disease continuum from healthy adults and SMD to MCI and AD. Further, we investigated multiple AD markers such as neurodegeneration, amyloid, APOE and cognition; and studied clinical progression over a long follow-up period. Finally, we used a powerful multivariate method able to condense large brain structural information into a single disease severity index with high clinical potential^24^,^25^. Some limitations should also be discussed. The sample size of the AD-like SMD subtype is small, which might have produced underpowered analyses, especially with regards to the cognitive variables and APOE ε4 status. However, this reduced size is to be expected when attempting to identify AD-like biological patterns within a clinical-normal group. AIBL is a convenience sample, and was initially enriched for APOE ε4 status, and so may not be entirely representative of the general population. Our analyses are based on subjective complaints constrained to a single item regarding memory. Although this is a frequent approach in the literature other studies have used inventories of memory complaints or composite scores derived from different tests or inventories^39^. Other authors have investigated the broader concept of SCD^4^, including other non-memory complaints such as executive functions, language and visuospatial abilities (e.g. refs 27 and 31). It would thus be of interest to study the AD-like pattern of atrophy in SCD.

In conclusion, SMD individuals evidenced intermediate cognitive performance between healthy adults without subjective complaints and MCI patients. Importantly, a subgroup of these was identified with an AD-like pattern of brain atrophy. This AD-like SMD evidenced increased amyloid burden, increased clinical severity as measured by the CDR-SOB, and 6.2 times higher likelihood to progress to MCI or dementia compared with healthy adults. Therefore, the disease severity index was able to identify asymptomatic individuals with a high risk to becoming symptomatic. Equally importantly, this index allowed the possible identification of SMD individuals with an aetiology other than neurodegenerative disease. Since this disease severity index has strong potential to be translated to the clinical workup^24^,^25^, the results of this study may have implications for identifying individuals where anti-dementia interventions should be initiated as early as possible (i.e. AD-like SMD). Individuals could also be identified where anti-dementia interventions would be definitely not indicated and another approach should be preferred (i.e. HC-like SMD). This method may thus have an impact for future clinical practice as well as selecting appropriate individuals for clinical trials and research. Finally, identification of sensitive objective cognitive measures for early detection of AD is an emerging field with high demand at the moment^21^,^31^. Results of the current study suggest that RCFT delayed recall may have potential to be considered in future assessment protocols.

## Methods

### Participants

Data were retrieved from the Australian Imaging Biomarkers and Lifestyle flagship study of ageing (AIBL)^40^, a large longitudinal study designed to discover potential biomarkers, cognitive characteristics as well as health and lifestyle factors that could determine later development of symptomatic AD. Those participants in the inception cohort receiving an MRI scan (N = 238) were included in the current study, comprising one hundred and fifty-five cognitively normal individuals (sixty-nine healthy controls and eighty-six SMD cases, see below), forty-five patients with MCI, and thirty-eight patients with AD. The study was approved by the institutional ethics committees of Austin Health, St Vincent’s Health, Hollywood Private Hospital and Edith Cowan University. Informed consent was obtained from all volunteers before participating in the study. All methods were performed in accordance with the relevant guidelines and regulations.

A full description of the cohort recruitment process including selection and diagnostic criteria is published elsewhere^40^. Briefly, allocation of individuals to one of the three diagnostic groups was undertaken by a clinical review panel comprised by two old age psychiatrists, a neurologist, a geriatrician and five neuropsychologists. Baseline classifications were discussed so as to ensure that diagnoses were made in a consistent manner according to internationally agreed criteria. AD diagnosis was based on the NINCDS-ADRDA criteria^41^ and MCI diagnosis was based on established criteria^29^,^42^. The criterion of cognitive impairment was operationalized as a score 1.5 SD or more below the age-adjusted mean using all the cognitive tests available (see below ‘Clinical and cognitive measures’ and a previous publication^40^ for further details). The criteria for the healthy controls and SMD required normal cognitive functioning as defined by cognitive scores no more than 1.5 SD below age-appropriate norms in all the cognitive tests available (see below ‘Clinical and cognitive measures’). For the purpose of this study, control individuals were further divided into SMD if they positively endorsed a question querying complaints of subjective memory decline (n = 86) or healthy controls if they denied such a complaint (n = 69). Memory complaints were elicited by the response to the question: “Do you have difficulties with your memory?”. Exclusion criteria for all the groups were a history of non-AD dementia, schizophrenia, bipolar disorder, significant current (but not past) depression (Geriatric Depression Scale^43^ - GDS - score above 5/15), Parkinson’s disease, cancer (other than basal cell skin carcinoma) within the last two years, symptomatic stroke, uncontrolled diabetes, obstructive sleep apnoea requiring continuous positive airway pressure, current regular alcohol use exceeding two standard drinks per day for women or four per day for men, or withdrawal of consent.

### Clinical and cognitive measures

A clinical interview and the fifteen-item GDS^43^ were used as screening instruments for all volunteers. The Clinical Dementia Rating (CDR)^44^, including the CDR-sum of boxes (CDR-SOB) was applied to assess clinical severity. The mini-mental state examination (MMSE)^45^ was used as a measure of global cognition. Detailed neuropsychological assessment included the California Verbal Learning Test–second edition (CVLT-II)^46^; verbal fluency (FAS for letter fluency; and animals and boys names for category fluency) from the Delis-Kaplan Executive Function System (D-KEFS)^47^; a thirty-item version of the Boston Naming Test (BNT)^48^; Digit Span (total over forward and backward tasks) and Digit Symbol-Coding subtests of the Wechsler Adult Intelligence Scale–Third edition (WAIS– III)^49^; the Stroop test–Victoria version^50^; and the Rey Complex Figure Test (RCFT)^51^. Anxious and depressive symptoms were further assessed with the Hospital Anxiety and Depression Scale (HADS)^52^.

### Magnetic resonance imaging

A 3D T1-weighted MPRAGE sequence was acquired with 1 × 1 x 1.2 mm^3^ resolution and the following parameters: repetition time/echo time/inversion time = 2300/2.98/900, flip angle = 9°, field of view = 240 × 256, 160 slices. Full brain and skull coverage was required for the MRI datasets and detailed quality control was carried out on all MR images according to previously published criteria^53^. Cortical reconstruction and volumetric segmentation were performed with the FreeSurfer 5.1.0 software package (http://surfer.nmr.mgh.harvard.edu/). Briefly, this procedure includes: (1) motion correction; (2) removal of nonbrain tissue^54^; (3) automated Talairach transformation; (4) segmentation of the subcortical structures^55^; (5) intensity normalization^56^; (6) tessellation of the gray matter white matter boundary; (7) automated topology correction^57^; (8) surface deformation following intensity gradients to optimally place the gray and/or white and gray and/or cerebrospinal fluid borders at the location where the greatest shift in intensity defines the transition to the other tissue class^58^,^59^; (9) registration to a spherical atlas^60^; (10) parcellation of the cerebral cortex into units based on gyral and sulcal structure^61^; and (11) creation of a variety of surface based data. Values of thickness and volume were calculated for sixty eight cortical regions^61^ and fifty one subcortical regions^55^, providing a total of one hundred eighty seven MRI measures. A measurement of total intracranial volume was estimated from the T1-weighted images with FreeSurfer 5.1.0 based on the linear transform to a standard space as described elsewhere^62^. This measurement of total intracranial volume was included in the models as a covariate to account for between-individual differences^63^.

### Positron emission tomography

Brain amyloid burden was investigated with PiB-PET. A 30-min acquisition scan starting 40 minutes after injection of ~370 MBq 11C-PiB was performed with Phillips Allegro™ PET cameras^11^. A preset in-house template of cortical regions of interest (ROIs) was applied to the PiB scan via placement on the subject’s co-registered MRI by an operator blind to the subject’s clinical status^11^. Co-registration of PiB to MRI was performed with SPM5^64^. The amyloid burden was expressed as the average of the mean of frontal, superior parietal, lateral temporal, lateral occipital, and anterior and posterior cingulate ROI activity per voxel divided by the cerebellar grey matter voxel activity and termed the SUVR. Baseline PiB standardized uptake values ratios (SUVR) was subsequently classified PiB-negative (SUVR < 1.5) or PiB-positive (SUVR ≥ 1.5) as previously reported^11^.

### Statistical analysis

One-way independent ANOVA was used for continuous and dummy variables, and the Chi-squared test for categorical variables. The Mann-Whitney U and the Kruskal-Wallis tests were used for ordinal and non-normally distributed continuous variables. All the analyses were replicated with ANCOVA in order to control for the effect of age, gender, education level and depressive symptomatology (i.e. HADS-D). MANOVA/MANCOVA were also used to test for between-group differences in a large number of cognitive variables (n = 14) while reducing the number of comparisons to one single test. Follow-up exploratory analyses were conducted to ascertain effects in individual cognitive variables. Mixed effects models (fixed and random effects) were used to analyse the interaction between a between-subjects factor (study group) and a within-subjects factor (time). The fixed-effect factors were study group, time, and the study group-by-time interaction. The random effect factor was the participants. Survival curves were created for studying group progression across five time points (baseline, 18, 36, 54, and 90 months follow-up). The Benjamini-Hochberg^65^ correction for multiple comparisons was applied in all the analyses, both across dependent variables and in post-hoc comparisons, using a p-value < 0.05 (two-tailed) as significant. Model assumptions were tested in all the cases by visual inspection of residuals and data distribution, as well as by inspecting the pertinent statistical parameters. All these analyses were performed using SPSS 22.0 (IBM Corp., Released 2011, Armonk, NY, USA).

The ‘disease severity index’ was calculated using the OPLS multivariate data analysis method as previously described in detail^16^. This method is included in the software package SIMCA (Umetrics AB, Umea, Sweden). Using the baseline MRI-derived measures (sixty eight cortical regions and fifty one subcortical regions) as input data, OPLS separates the systematic variation in data into two blocks: predictive and orthogonal. The first component of the model is predictive and includes information related to class separation (e.g. AD *vs.* healthy controls). The orthogonal components in the model are related to other variation in the data not related to the actual problem, such as within class variation. The predictive component is ascribed the goodness of prediction (Q^2^) value which defines how reliable a model predicts new data. A model with a Q^2^ value > 0.05 is regarded statistically significant, and > 0.5 is regarded a good model^66^. In the current study, an OPLS classification model was trained using a 7-fold cross validation method^67^ to separate AD patients from healthy controls. We have previously used 7-fold cross validation in several studies (e.g. refs 16, 24, 25 and 63). The cross validated model was used to estimate Q^2^ and report sensitivity and specificity values. Then, the SMD individuals were projected onto this model as unseen data in order to discriminate between SMD individuals with an AD-like (AD-like) pattern of brain atrophy from SMD patients with an HC-like pattern of brain atrophy. The OPLS model assigns a score to each SMD subject (‘disease severity index’) where a score close to one represents AD-like pattern and a score close to zero represents HC-like pattern. The cut-off value for classifying the SMD individuals as AD-like or HC-like was set to 0.5, as previously done for the same purpose in MCI patients^24^.

Finally, vertex analyses across the cortical mantle were conducted using the FreeSurfer software to investigate differences in cortical thickness between AD-like and HC-like SMD. A general linear model was fitted at each vertex using cortical volume as the dependent variable and diagnostic group as the independent variable. Results were tested against an empirical null distribution of maximum cluster size across five thousand iterations. Monte Carlo Null-Z simulations were used with a cluster-forming threshold of p ≤ 0.05 (two-sided), yielding clusters corrected for multiple comparisons across the cortical mantle.

## Additional Information

**How to cite this article:** Ferreira, D. *et al*. A ‘Disease Severity Index’ to identify individuals with Subjective Memory Decline who will progress to mild cognitive impairment or dementia. *Sci. Rep.* **7**, 44368; doi: 10.1038/srep44368 (2017).

**Publisher's note:** Springer Nature remains neutral with regard to jurisdictional claims in published maps and institutional affiliations.

## Figures and Tables

**Table 1:** Demographic-clinical characteristics of study groups.

	HC (n = 69)	SMD (n = 86)	MCI (n = 45)	AD (n = 38)	Uncorrected p-value	BH corrected p-value
Age	72.7 (7.2)	72.8 (7.1)	76.4 (7.3)	72.6 (8.8)	0.057	0.150
Gender, % female	51	52	51	58	0.906	0.906
Education level	3 (1–4)	2 (1–4)	2 (0–4)	2 (1–4)^a^	**0.014**	0.056
MMSE	29.0 (1.1)	28.6 (1.3)	27.2 (2.2)^a,b^	20.4 (5.5)^a,b.c^	**0.001**	**<0.001**
CDR-SOB	0.01 (0.06)	0.05 (0.15)	1.05 (0.78)^a,b^	4.95 (2.95)^a,b,c^	**<0.001**	**<0.001**
HADS-D	2.1 (2.0)	3.0 (2.2)	3.4 (2.1)^a^	4.8 (4.7)^a^	**0.003**	**0.015**
HADS-A	3.7 (2.8)	4.8 (3.0)	4.6 (2.4)	5.8 (4.4)	0.075	0.150
APOE, % ε4 carriers	46	38	58	75^a,b^	**0.002**	**0.012**
0 ε4 alleles, n	37	53	19	9	—	—
1 ε4 allele, n	30	31	21	19	—	—
2 ε4 alleles, n	2	2	5	8	—	—

Values in the table represent the mean and standard deviation except for gender and APOE, where the percentage is shown; for education level, where median and minimum–maximum is shown (Education level: 0 = 0–6 years, 1 = 7–8 years, 2 = 9–12 years, 3 = 13–15 years, 4 = 15+); and for the number of APOE ε4 alleles, where count is shown. The same results were obtained for MMSE and CDR-SOB when controlling for age, gender, education level and HADS-D. ^a^Significantly different from HC; ^b^significantly different from SMD; ^c^significantly different from MCI. HC = healthy controls; SMD = subjective memory decline; MCI = Mild Cognitive Impairment; AD = Alzheimer’s disease; BH = Benjamini-Hochberg; MMSE = Mini-Mental State Examination; CDR-SOB = Clinical Dementia Rating–Sum of Boxes; HADS = Hospital Anxiety and Depression Scale (D = depression subscale score; A = anxiety subscale score); APOE = apolipoprotein E e4 allele.

**Table 2:** Cognitive performance across study groups.

		HC (n = 69)	SMD (n = 86)	MCI (n = 45)	AD (n = 38)	MANOVA/MANCOVA*	Post-hoc ANOVA/ANCOVA	
							Uncorrected p-value	BH corrected p-value
Stroop-colours	M(SD)	13.8 (3.2)	14.6 (3.1)^a^	16.6 (5.3)^a^	25.2 (13.2)^a,b,c^	Multivariate effect: <0.001 Post-hoc paired tests: HC *vs.* SMD p = 0.015 *^4^ HC *vs.* MCI p < 0.001 HC *vs.* AD p < 0.001 SMD *vs.* MCI p < 0.001 SMD *vs.* AD p < 0.001 MCI *vs.* AD p = 0.001 *^5^	<0.001	<0.001
	Z-score	−0.1	0.4	0.9	3.0			
Stroop-words	M(SD)	18.0 (4.1)	19.3 (4.9)^a^*^1^	24.8 (9.0)^a,b^	38.4 (20.9)^a,b,c^		<0.001	<0.001
	Z-score	−0.1	0.4	1.3	3.1			
Stroop-interference	M(SD)	33.1 (9.2)	33.9 (11.4)	47.9 (21.1)^a,b^	92.2 (87.6)^a,b,c^		<0.001	<0.001
	Z-score	−0.3	−0.2	0.4	2.8			
Digit Symbol-Coding	M(SD)	59.8 (13.4)	55.8 (12.3)	49.1 (15.5)^a,b^*^2^	32.1 (14.8)^a,b,c^		<0.001	<0.001
	Z-score	0.6	0.3	0.1	−1.1			
Digit Span	M(SD)	18.3 (3.8)	17.7 (3.8)	16.4 (3.4)^a†^	13.4 (4.3)^a,b,c^		<0.001	<0.001
	Z-score	0.8	0.6	0.3	−0.4			
CVLT-II Learning	M(SD)	52.3 (10.9)	50.2 (10.7)	31.2 (8.8)^a,b^	20.7 (8.7)^a,b,c^		<0.001	<0.001
	Z-score	1.3	1.1	−0.9	−2.2			
CVLT-II Delayed recall	M(SD)	12.1 (2.9)	11.3 (3.3)	4.0 (3.2)^a,b^	1.0 (2.0)^a,b,c^		<0.001	<0.001
	Z-score	1.0	0.8	−1.5	−2.6			
CVLT-II Recognition	M(SD)	14.5 (1.6)	15.0 (1.5)	12.7 (2.8)^a,b^	11.6 (2.5)^a,b,c^*^3^		<0.001	<0.001
	Z-score	0	0.3	−1.0	−1.8			
RCFT Copy	M(SD)	31.9 (3.0)	31.2 (3.5)	27.8 (6.2)^a,b^	21.1 (10.7)^a,b,c^		<0.001	<0.001
	Z-score	−0.2	−0.4	−1.3	−4.2			
RCFT Delayed recall	M(SD)	17.1 (5.4)	16.1 (6.0)	8.9 (5.5)^a,b^	2.7 (3.0)^a,b,c^		<0.001	<0.001
	Z-score	1.0	0.7	−0.8	−2.5			
RCFT Recognition	M(SD)	20.2 (1.9)	20.4 (2.2)	18.6 (2.0)^a,b^	15.9 (2.6)^a,b,c^		<0.001	<0.001
	Z-score	0.3	0.4	−0.8	−2.6			
BNT	M(SD)	28.6 (1.6)	27.8 (2.0)^a^*^1^	25.1 (4.8)^a,b^	20.9 (7.4)^a,b,c^		<0.001	<0.001
	Z-score	0.9	0.8	0.1	−1.0			
VF-letter	M(SD)	40.9 (11.8)	40.4 (12.6)	33.1 (12.4)^a,b^	27.1 (14.5)^a,b^		<0.001	<0.001
	Z-score	0.6	0.6	−0.1	−0.7			
VF-category	M(SD)	41.7 (8.0)	36.7 (7.3)^a^	31.2 (9.0)^a,b^	20.9 (9.2)^a,b,c^		<0.001	<0.001
	Z-score	1.1	0.4	−0.2	−1.5			

Performance is measured as time in Stroop and number of correct responses in the other cognitive tests. Z-scores were calculated using age- and education-corrected Australian normative values. ^a^Significantly different from HC; ^b^significantly different from SMD; ^c^significantly different from MCI. *Results from the MANCOVA are marked with *. *^1^SMD *vs.* HC non-significant when controlling for age, gender, education level and HADS-D; *^2^MCI *vs.* SMD non-significant when controlling for age, gender, education level and HADS-D; *^3^AD *vs.* MCI becomes significant (p = 0.008 after Benjamini-Hochberg’s correction) when controlling for age, gender, education level and HADS-D; *^4^p = 0.020 after Benjamini-Hochberg’s correction when controlling for age, gender, education level and HADS-D; *^5^p = 0.002 after Benjamini-Hochberg’s correction; ^†^MCI *vs.* HC p = 0.055 after Benjamini-Hochberg’s correction. M(SD) = mean (standard deviation); HC = healthy controls; SMD = subjective memory decline; MCI = Mild Cognitive Impairment; AD = Alzheimer’s disease; BH = Benjamini-Hochberg; CVLT-II = California Verbal Learning Test-Second edition; RCFT = Rey Complex Figure Test; BNT = Boston Naming Test; VF = Verbal Fluency.

**Table 3:** Demographic-clinical characteristics of the SMD subtypes.

	HC-like SMD (n = 75)	AD-like SMD (n = 11)	Uncorrected p-value	BH corrected p-value
Age	72.5 (6.8)	75.3 (8.8)	0.292	0.985
Gender, % female	56	27	0.076	0.528
Education level	2 (1–4)	3 (1–4)	0.973	0.985
MMSE	28.7 (1.2)	27.8 (1.7)	0.088	0.528
CDR-SOB	0.0 (0.1)	0.2 (0.3)	**0.001**	**0.008**
HADS-D	2.9 (2.2)	3.3 (3.1)	0.985	0.985
HADS-A	4.8 (3.0)	5.4 (3.3)	0.658	0.985
APOE, % ε4 carriers	36	55	0.243	0.985
0 ε4 alleles, n	48	5	—	—
1 ε4 allele, n	26	5	—	—
2 ε4 alleles, n	1	1	—	—

Values in the table represent the mean and standard deviation except for gender and APOE where the percentage is shown; for education level, where median and minimum–maximum is shown (Education level: 0 = 0–6 years, 1 = 7–8 years, 2 = 9–12 years, 3 = 13–15 years, 4 = 15+); and for the number of APOE ε4 alleles, where count is shown. The same results were obtained for MMSE and CDR-SOB when controlling for age, gender, education level and HADS-D. SMD = subjective memory decline; HC-like SMD = SMD individuals evidencing a healthy-like pattern of brain atrophy; AD-like SMD = SMD individuals evidencing an Alzheimer’s disease-like pattern of brain atrophy; BH = Benjamini-Hochberg; MMSE = Mini-Mental State Examination; CDR-SOB = Clinical Dementia Rating–Sum of Boxes; HADS = Hospital Anxiety and Depression Scale (D = depression subscale score; A = anxiety subscale score); APOE = apolipoprotein E e4 allele.

**Table 4:** Cognitive performance in the SMD subtypes.

		HC-like SMD (n = 75)	AD-like SMD (n = 11)	MANOVA/MANCOVA*	Post-hoc ANOVA/ANCOVA	
					p-value	BH corrected p-value
Stroop-colours	M(SD)	15.0 (3.3)	16.6 (4.5)	Multivariate effect: 0.140	0.341	0.880
	Z-score	0.4	0.4			
Stroop-words	M(SD)	19.6 (4.9)	22.6 (4.4)		0.039*^1^	0.468*^1^
	Z-score	0.3	0.8			
Stroop-interference	M(SD)	33.5 (11.5)	40.5 (13.0)		**0.033**	0.429
	Z-score	-0.3	0.1			
Digit Symbol-Coding	M(SD)	57.0 (12.0)	47.8 (12.8)		**0.043**	0.473
	Z-score	0.4	−0.1			
Digit Span	M(SD)	17.7 (4.0)	17.5 (1.7)		0.735	0.880
	Z-score	0.6	0.5			
CVLT-II Learning	M(SD)	50.5 (10.3)	48.4 (13.8)		0.493	0.880
	Z-score	1.0	1.1			
CVLT-II Delayed recall	M(SD)	11.5 (3.1)	10.0 (4.0)		0.212	0.880
	Z-score	0.8	0.5			
CVLT-II Recognition	M(SD)	15.0 (1.6)	15.5 (0.8)		0.302	0.880
	Z-score	0.3	0.6			
RCFT Copy	M(SD)	31.2 (3.7)	31.5 (2.2)		0.740	0.880
	Z-score	−0.4	−0.3			
RCFT Delayed recall	M(SD)	16.7 (6.1)	12.0 (4.0)		0.017*^2^	0.238*^2^
	Z-score	0.8	−0.1			
RCFT Recognition	M(SD)	20.5 (2.2)	20.4 (1.8)		0.684	0.880
	Z-score	0.4	0.4			
BNT	M(SD)	27.6 (2.1)	27.5 (2.7)		0.880	0.880
	Z-score	0.8	0.7			
VF-letter	M(SD)	40.3 (12.7)	41.7 (12.2)		0.665	0.880
	Z-score	0.6	0.7			
VF-category	M(SD)	36.9 (7.7)	35.3 (4.2)		0.420	0.880
	Z-score	0.5	0.3			

Performance is measured as time in Stroop and number of correct responses in the other cognitive tests. Z-scores were calculated using age- and education-corrected Australian normative values. *Results from the MANCOVA are marked with *. *^1^p = 0.055 (uncorrected) and p = 0.440 (after Benjamini-Hochberg’s correction) when controlling for age, gender, education level and HADS-D; *^2^ p = 0.004 (uncorrected) and p = 0.040 (after Benjamini-Hochberg´s correction) when controlling for age, gender, education level and HADS-D. M(SD) = mean (standard deviation); SMD = subjective memory decline; HC-like SMD = SMD individuals evidencing a healthy-like pattern of brain atrophy; AD-like SMD = SMD individuals evidencing an Alzheimer’s disease-like pattern of brain atrophy; BH = Benjamini-Hochberg; CVLT-II = California Verbal Learning Test-Second edition; RCFT = Rey Complex Figure Test; BNT = Boston Naming Test; VF = Verbal Fluency.

**Table 5:** Diagnosis at follow-up and longitudinal changes in MMSE and CDR-SOB.

		HC	HC-like SMD	AD-like SMD	MCI	AD	p-value
Diagnosis at follow-up	HC	35 (52%)	**11 (15%)**	**0**	1 (2%)	0	0.175
	SMD	25 (37%)	50 (68%)	3 (27%)	2 (5%)	0	**—**
	MCI	**7 (10%)**	**9 (12%)**	**0**	14 (33%)	0	0.474
	AD	**0**	**1 (1%)**	**6 (55%)**^**a,b**^	**17 (40%)**^**a,b**^	19 (68%)	<0.001
	PD	0	0	0	1 (1%)	0	**—**
	Died	**1 (1%)**	**3 (4%)**	**2 (18%)**	**8 (19%)**	**9 (32%**)^**a,b**^	<0.001
MMSE		**—**	**0.08**	**−0.65**	**−1.28**	**−8.16**	<0.001
CDR SOB		**—**	**−0.14**	**−0.04**	**0.84**	**4.53**	<0.001

ANCOVA was performed for some contrasts of interest defined a-priori (indicated in bold), and corresponding p-values are reported in the table. Age, gender, education level and HADS-D were in included as covariates. Values in the table represent count (and percentage) for diagnosis at follow-up, and estimated change over time (HC as reference) in MMSE and CDR-SOB from the mixed effects models. MMSE and CDR-SOB include follow-up up to 54 months due to numerous missing data for these variables at 72 and 90 months. ^a^Significantly different from HC; ^b^significantly different from HC-like SMD. HC = healthy controls; SMD = subjective memory decline; HC-like SMD = SMD individuals evidencing a healthy-like pattern of brain atrophy; AD-like SMD = SMD individuals evidencing an Alzheimer’s disease-like pattern of brain atrophy; MCI = Mild Cognitive Impairment; AD = Alzheimer’s disease; PD = Parkinson’s disease with dementia; MMSE = Mini-Mental State Examination; CDR-SOB = Clinical Dementia Rating–Sum of Boxes.
